# The rs738409 polymorphism of the PNPLA3 gene is associated with hepatic steatosis and fibrosis in Brazilian patients with chronic hepatitis C

**DOI:** 10.1186/s12879-017-2887-6

**Published:** 2017-12-19

**Authors:** Caroline Manchiero, Arielle Karen da Silva Nunes, Mariana Carvalheiro Magri, Bianca Peixoto Dantas, Celso Carmo Mazza, Antonio Alci Barone, Fátima Mitiko Tengan

**Affiliations:** 1 Medical Research Laboratory for Virus (Laboratório de Investigação Médica por Vírus - LIM47) of the Clinics Hospital, School of Medicine, University of São Paulo (Universidade de São Paulo - USP), São Paulo, Brazil; 2 Department of Infectious and Parasitic Diseases, School of Medicine, University of São Paulo, São Paulo, Brazil

**Keywords:** Hepatitis C, PNPLA3, Steatosis, Fibrosis, Polymorphism rs738409, TM6SF2, Polymorphism rs58542926, Brazil

## Abstract

**Background:**

Prospective studies have shown that 80% of acute hepatitis C virus (HCV) cases progress to chronic infection; approximately 10-20% of patients with these conditions will develop liver cirrhosis within 2 to 3 decades, and 1-5% will develop liver cancer. Some studies have indicated that the rs738409 polymorphism of the PNPLA3 gene is associated with steatosis and the progression of advanced fibrosis. This study assessed the contribution of the PNPLA3 rs738409 polymorphism with regard to the steatosis and degree of liver fibrosis in Brazilian patients diagnosed with chronic hepatitis C.

**Methods:**

A total of 290 patients were evaluated at the Clinics Hospital of the School of Medicine, University of São Paulo, between 2010 and 2015. The inclusion criteria were age ≥ 18 years and positive anti-HCV antibody and HCV RNA tests. The participants were evaluated based on medical consultation, blood tests, and liver biopsies conducted before specific antiviral therapies were applied. The associations between the rs738409 PNPLA3 gene polymorphism and steatosis and advanced fibrosis were tested under a recessive inheritance model using logistic regression analysis, including age, gender, BMI, ethnicity/color, HOMA-IR, alcohol intake, HCV genotype 3, and the rs58542926 TM6SF2 gene polymorphism as covariates.

**Results:**

The mean age of the patients was 54.9 years old (range, 28 to 82 years), and 124 (42.8%) patients were male; 226 (77.9%) were white, 43 (14.8%) were *pardo,* and 21 (7.2%) were black Brazilians. Of the patients included in this study, 133 (45.9%) presented with the CC genotype, 63 (21.7%) with the CG genotype, and 94 (32.4%) with the GG genotype of the PNPLA3 gene I148M variant. We observed that the associations between PNPLA3 rs738409 GG genotype and steatosis was significant (OR: 2.16; 95% CI 1.26-3.72). The same genotype was associated to advanced fibrosis too (OR:2.64; 95% CI 1.26-5.53).

**Conclusions:**

Associations between the rs738409 polymorphism of the PNPLA3 gene genotype GG and hepatic steatosis and advanced fibrosis were observed. Studies are still needed to clarify the influence of these polymorphisms on hepatic steatosis and degree of fibrosis among individuals diagnosed with chronic hepatitis C.

**Electronic supplementary material:**

The online version of this article (10.1186/s12879-017-2887-6) contains supplementary material, which is available to authorized users.

## Background

The hepatitis C virus (HCV) has a worldwide distribution, occurring among individuals of all ages and ethnicities and across various regions of the world. HCV infection affects 2.2-3.0% of all people in the world (130-170 million people), with greater rates in Africa and Asia [1]. The estimated prevalence in Brazil is 1.4%, with 2,609,670 people infected [1]. Prospective studies have shown that 80% of acute hepatitis C cases progress to chronic infection. Approximately 10-20% of these cases develop complications of chronic liver disease, such as liver cirrhosis within 2 to 3 decades, and 1-5% will develop liver cancer [2, 3].

Clinical factors (e.g., advanced age, male gender, elevated body mass index [BMI], and alcohol consumption [> 30-50 g/day]), histological factors (e.g., steatosis), viral factors (primarily genotype 3), and HCV-human immunodeficiency virus (HIV) coinfection are associated with a greater risk of fibrosis progression. However, even when considered together, these factors have a predictive value of less than 30% [4].

The genetic factors that influence the evolution of hepatitis C are being studied, including the rs738409 polymorphism of the patatin-like phospholipase domain containing 3 (PNPLA3) gene. This polymorphism, located on chromosome 22, is characterized by the replacement of isoleucine with methionine at position 148 (I148M) due to exchange of a C nucleotide by a G (C>G) and has been strongly associated with liver fat content and high levels of serum alanine aminotransferase and aspartate aminotransferase [5]. Studies of Caucasian patients suggest that the rs738409 polymorphism is associated with steatosis and the progression of advanced fibrosis [6–8].

More recently, a single-nucleotide polymorphism encoding the E167K variant of the transmembrane six superfamily member 2 gene (TM6SF2) was determined to be independently associated with liver steatosis [9, 10] and necroinflammation [10] in chronic hepatitis C patients.

The aim of this study was to evaluate the contribution of the PNPLA3 rs738409 genetic variant to steatosis and the degree of liver fibrosis in Brazilian patients diagnosed with chronic hepatitis C. The secondary objective was to evaluate the role of the gene TM6SF2 E167K variant in the occurrence of the same outcomes.

## Methods

### Patient selection

A total of 290 patients were evaluated at the outpatient clinic of infectious diseases in the Clinics Hospital of the School of Medicine, University of São Paulo (Hospital das Clínicas da Faculdade de Medicina da Universidade de São Paulo; HCFMUSP). The inclusion criteria were age ≥ 18 years and positive anti-HCV antibody (3rd generation ELISA) and HCVRNA tests (Cobas Amplicor HCV Monitor Test, Roche Diagnostics, Branchburg, New Jersey, USA). Patients co-infected with hepatitis B virus (HBV) or HIV or who had received previous HCV treatment were excluded.

**Fig. 1:**
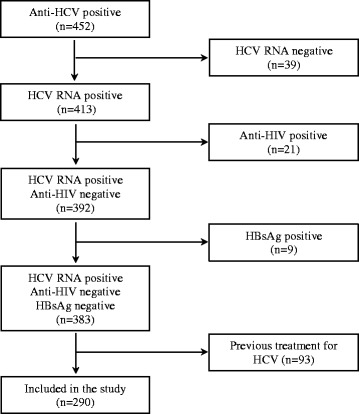
Patient selection flowchart for the present study

In the period from 01/01/2010 to 31/12/2015, 452 patients with anti-HCV positive tests were attended at the outpatient clinic of infectious diseases in the Clinics Hospital of the HCFMUSP. Of these, 413 were positive for HCV RNA, and 39 were negative for HCV RNA (Fig. 1). Of the 413 positive for HCV RNA, 21 patients were anti-HIV antibody-positive, and 9 were HBsAg-positive. Of the remaining 383 patients, 93 reported previous treatment for hepatitis C. Therefore, 290 patients with chronic hepatitis C were included in the study. The participants were evaluated based on medical consultation, blood tests, and liver biopsies conducted before specific antiviral therapies were applied. The ethics committee of HCFMUSP approved this study and all study participants provided written consent.

### Data collection

The epidemiological factors collected included gender, age, ethnicity/color, alcohol consumption, and body mass index (BMI). Alcohol consumption was divided into two categories: ≤ 20 g/day or > 20 g/day. BMI was calculated as the ratio between weight in kg and height (meters squared). Insulin resistance was estimated using the HOMA-IR (homeostasis model assessment) value and was defined by the equation fasting insulin (μU/mL) x fasting glucose (mmol/L)/22.5.

In the present study, Brazilians were classified according to their own self-identification of ethnicity/color: African Brazilians, self-identified as "black"; European Brazilians, self-identified as "white"; a mixture of one or more ethnicities, self-identified as "*pardo*"; Brazilian Asian, self-identified as "yellow"; and indigenous Brazilian, self-identified as "indigenous." In Brazil, the term most used to define multiracial people is *pardo* (brown), which is the result of a mixture of various ethnicities [11]. The term has been officially adopted by the Instituto Brasileiro de Geografia e Estatística (BGE - Brazilian Institute of Geography and Statistics), which conducts the Brazilian demographic census [11].

Histological abnormalities were scored using the Metavir system. Liver fibrosis was rated using a 5-point scale, where F0 = no fibrosis, F1 = portal fibrosis without septa, F2 = rare septa, F3 = numerous septa without cirrhosis, and F4 = cirrhosis. Necroinflammatory activity was rated using a 4-point scale, where A = no histological activity, A1 = mild activity, A2 = moderate activity, and A3 = severe activity [12]. Steatosis was evaluated based on the percentage of hepatocytes containing fat droplets and was classified as 0 (<5%), 1 (5-33%), 2 (34-66%), or 3 (> 66%).

Genotyping was conducted using the Versant ® HCV Genotype 2.0 (LiPA) test (Imunogenetics, Ghent, Belgium) according to the manufacturer's instructions.

### PNPLA3 and TM6SF2 genotyping

The extraction and purification of genomic DNA from the serum were conducted using a PureLink Genomic DNA Mini kit (Invitrogen/Life Technologies, Carlsbad, California, USA) according to the manufacturer’s instructions.

After DNA extraction, the samples were subjected to a PCR-RFLP reaction to analyze the rs738409 polymorphism of the PNPLA3 gene where the dominant homozygous genotype is CC, the heterozygous genotype is CG and the recessive / mutated homozygote is GG. The following primers were used: F: 5’-TGGGCCTGAAGTCCGAGGGT-3’ and R: 5’-CCGACACCAGTGCCCTGCAG-3’. To determine the rs738409 (C>G) polymorphism genotype, the restriction endonuclease BtsCI (BtsCI, 20,000 U/ml, New England BioLabs, Massachusetts, USA) was used based on the descriptions of Dutta [13]. The products of the restriction enzyme digestion were subjected to electrophoresis in a 2% agarose gel with 6 μL of *Diamond*™ Nucleic Acid Dye (Promega, Fitchburg, Wisconsin, USA) in a horizontal electrophoretic tank containing TBE buffer (0.5X concentration) with a constant current of 110 V for 1 hour and 15 minutes. The resulting bands were visualized under ultraviolet (UV) light using a UVIdoc HD2 gel documentation system (Uvitec Cambridge, Cambridge, UK). The images were recorded to digital files.

Regarding the rs58542926 polymorphism (C>T) of the TM6SF2 gene, samples were subjected to a real-time PCR using the system “TaqMan genotyping assay”, dbSNP rs58542926 assay C_89463510_10, #4351379 (Applied Biosystems, Foster City, CA, USA) on a StepOnePlus™ (96-well) Real-Time PCR System (Applied Biosystems, Foster City, CA, USA). The assay was standardized in a final volume of 25 μL: 12.5 μL of TaqMan MasterMix (Applied Biosystems, Foster City, CA, USA), 0.63 μL of Genotyping Assay 40X (Applied Biosystems, Foster City, CA, USA), 0.63 μL of ultrapure water (Promega, Fitchburg, Wisconsin, USA), and 11.25 μL of genomic DNA. The cycling was as follows: 95°C for 10 minutes, followed by 50 cycles of 95°C for 15 seconds and 60°C for 1 minute, and finally, 60°C for 1 minute. The interpretation of genotypes was given by CC, CT, and TT, where T is the mutated allele.

### Genetic inheritance model

The role of the polymorphism rs738409 of the PNPLA3 gene on steatosis and fibrosis was investigated using the recessive genetic inheritance model, comparing patients with the homozygous G allele (GG genotype) with patients with one or no copy of the G allele (CG or CC). The model of recessive genetic inheritance was chosen, based on previous results of studies on the association between genotypes of polymorphism rs738409 PNPLA3 and fibrosis in patients with nonalcoholic fatty liver disease (NAFLD) and Hepatitis C performed by other authors [7, 14].

In addition, in order to better understand the risk associated with the rs738409 polymorphism of the PNPLA3 gene, we compared, as an additional analysis, patients with the G allele (CG+GG) and patients with the genotype CC (dominant model) and patients with GG genotype and patients with genotype CC (additive model).

Finally, as a secondary objective, we evaluated the role of the gene TM6SF2 E167K variant in the occurrence of the same outcomes.

### Statistical analyses

As a primary objective we assessed the effect of PNPLA3 on steatosis and degree of liver fibrosis in Brazilian patients diagnosed with chronic hepatitis C using a recessive (genotype CG+CC vs GG.) inheritance genetic model and as an additional analysis we evaluate the same effects under a dominant (genotype CC vs. CG+GG) and additive (genotype CC vs. GG) genetic model. As a secondary objective, we evaluated the role of the TM6SF2 gene E167K variant in the occurrence of the same outcomes.

The associations between the rs738409 PNPLA3 gene polymorphism and steatosis and advanced fibrosis were tested using logistic regression analysis, including age, gender, BMI, ethnicity/color, HOMA-IR, alcohol intake, HCV genotype 3, and the rs58542926 TM6SF2 gene polymorphism as covariates. The effect on advanced fibrosis was evaluated by comparing patients with no fibrosis, mild fibrosis, or moderate fibrosis (F0-F1-F2) with those with severe fibrosis or cirrhosis (F3-F4). The presence of steatosis was studied as a qualitative variable (< 5% versus ≥ 5%). To perform the analyses, the software IBM-SPSS for Windows version 20.0 was used; for data tabulation, the software Microsoft Excel 2003 was used. The tests were performed with a significance level of 5%.

## Results

The characteristics of the patients with chronic hepatitis C included in the study are shown in Table 1. The mean age of the patients was 54.9 years old (range, 28 to 82 years), and 124 (42.8%) patients were male. Of the 290 patients included in the study, 226 (77.9%) were white, 43 (14.8%) were *pardo,* and 21 (7.2%) were black Brazilians. The mean BMI was 27 kg/m^2^; 107 (36.9%) and 66 (22.8%) of these patients were classified as overweight (25.0-29.9 kg/m^2^) and obese (≥30 kg/m^2^), respectively. Of the 290 patients, 133 (45.9%) presented with the CC genotype, 63 (21.7%) presented with the CG genotype, and 94 (32.4%) presented with the GG genotype of PNPLA3 polymorphism. For the TM6SF2, 29 (10.0%) presented with CT genotype, and 261 (90.0%) presented with the CC genotype.

**Table 1:** Baseline characteristics of patients included in the study

Characteristics	Total
*N*	290
Age (mean±SD)	54.9±12.1
Male gender	124 (42.8%)
Ethnicity/color	
White	226 (77.9%)
*Pardo*	43 (14.8%)
Black	21 (7.2%)
Alcohol > 20 g/day	55(19.3%)
BMI (mean±SD)	27.1±6.1
Glucose (mean±SD)	93.1±49.4
Insulin (mean±SD)	15.2±32.2
ALT (mean±SD)	62.3±49.4
AST (mean±SD)	49.8±38.1
Total cholesterol (mean±SD)	173.9±39.8
LDL-C (mean±SD)	95.5±32.8
HDL-C (mean±SD)	55.3±18.5
VLDL-C (mean±SD)	23.9±20.9
Triglycerides (mean±SD)	109.3±66.8
Hepatic steatosis ≥ 5%	143 (49.3%)
Liver fibrosis (Metavir ≥ F3)	58 (20%)
HCV genotype 3	45 (15.7%)
PNPLA3 rs738409	
CC	133 (45.9%)
CG	63 (21.7%)
GG	94 (32.4%)
TM6F2 rs58542926	
CC	261 (90%)
CT	29 (10%)

*Abbreviations*: *BMI* body mass index, *ALT* alanine aminotransferase, *AST* aspartate aminotransferase, *LDL-C* low-density lipoprotein-cholesterol, *HDL-C* high-density lipoprotein-cholesterol, *VLDL* very-low-density lipoprotein-cholesterol

The most frequent GG genotype of the PNPLA3 gene was found in white Brazilians (35.4%), followed by *pardo* (23.3%) and black (19.1%) Brazilians. Thus, the highest frequency of the PNPLA3 allele G was found in white Brazilians (45.6%), followed by black (35.7%) and *pardo* (34.9%) Brazilians; details of the clinical characteristics stratified by ethnicity and genotype polymorphism at rs738409 PNPLA3 are shown in Additional file 1.

After adjusting for age, gender, BMI, HOMA-IR, ethnicity/color, alcohol use, HCV genotype 3, and TM6SF2 genotypes, the association between PNPLA3 rs738409 GG genotype and steatosis remained significant in the recessive (*p*=0.01) model (Table 2).

**Table 2:** Analysis of the association between genotype polymorphism at rs738409 PNPLA3 and steatosis and advanced liver fibrosis in patients with HCV infection according to the univariate and multivariate analysis in the recessive model

Variables	n^a^/N^b^ (%)	n^a^/N^b^ (%)	Univariate analysis			Multivariate analysis		
	No steatosis	Steatosis	OR^c^	95% CI	*P*	OR	95% CI	*P*
PNPLA3 genotype								
CC/CG	108/147 (74.1)	88/143 (61.5)	1.00			1.00		
GG	38/147 (25.9)	55/143 (38.5)	1.73	(1.05 – 2.84)	0.03	2.16	(1.26 – 3.72)	0.01
Age (years)	52.2±11.7	57.6±11.8	0.03	(0.01 – 0.06)	0.00	1.04	(1.02 – 1.06)	0.00
Sex								
Female	78/147 (53.1)	88/143 (61.5)	1.00			1.00		
Male	69/147 (46.9)	55/143(38.6)	1.41	(0.88 – 2.25)	0.14	0.62	(0.35 – 1.08)	0.09
BMI (kg/m2)	26±3.8	28.1±7.6	0.07	(0.03 – 0.13)	0.00	1.10	(1.04 – 1.18)	0.00
Ethnicity/color								
White	108/147 (73.5)	118/143 (82.5)	1.00			1.00		
Pardo	28/147 (19.0)	15/143(10.5)	0.64	(0.33 – 1.24)	0.19	0.77	(0.36 – 1.66)	0.50
Black	11/147 (7.5)	10/143 (7.0)	1.34	(0.53 – 3.23)	0.55	0.71	(0.25 – 2.05)	0.53
HOMA-IR	3.1±8.2	3.8±3.7	0.02	(0.01 – 0.07)	0.38	0.99	(0.95 – 1.03)	0.72
Alcohol								
<20 g/day	113/147 (76.9)	117/143 (81.8)	1.00			1.00		
>20 g/day	31/147 (21.1)	24/143(16.8)	0.75	(0.41 – 1.36)	0.35	1.38	(0.77 – 2.48)	0.29
HCV genotype								
Non 3	130/147 (88.4)	111/143 (77.6)	1.00			1.00		
3	15/147 (10.2)	29/143(20.3)	2.34	(1.19 – 4.57)	0.01	2.15	(1.02 – 4.53)	0.04
TM6SF2 (genotype								
CC	135/147 (91.8)	126/143 (88.1)	1.00			1.00		
CT	12/147 (8.2)	17/143(11.9)	0.95	(0.44 – 2.05)	0.90	2.28	(0.93 – 5.61)	0.07
	Mild fibrosis (F0-F1-F2)	Advanced Fibrosis (F3-F4)						
PNPLA3 genotype								
CC/CG	162/232 (69.8)	34/58 (58.6)	1.00			1.00		
GG	70/232 (30.2)	24/58(41.4)	1.63	(1.15 – 2.95)	0.01	2.64	(1.26 – 5.53)	0.01
Age (years)	53.4±13.3	60.7±9.2(84.5)	0.05	(0.02 – 0.08)	0.00	1.08	(1.04 – 1.11)	0.00
Sex								
Female	145/232 (62.5)	21/58 (36.2)	1.00			1.00		
Male	87/232 (37.5)	37/58(63.8)	2.93	(1.61 – 5.33)	0.00	1.06	(1.03 – 1.09)	0.00
BMI (kg/m2)	27.1±6.4	26.8±4.8	1.00	(0.95 – 1.05)	0.71	1.03	(0.97 – 1.09)	0.37
Ethnicity/color								
White	183/232 (78.9)	43/58 (74.1)	1.00			1.00		
Pardo	16/232 (6.9)	10/58(17.3)	1.36	(0.62 – 2.99)	0.43	2.46	(0.93 – 6.48)	0.07
Black	33/232 (14.2)	5/58(8.6)	2.25	(0.85 – 5.94)	0.10	1.76	(0.45 – 6.93)	0.42
HOMA-IR	3.2±6.7	4.6±4.5	0.02	(0.01 – 1.02)	0.21	1.03	(0.99 – 1.08)	0.15
Alcohol								
<20 g/day	187/232 (80.6)	43/58 (74.1)	1.00			1.00		
>20 g/day	40/232 (17.2)	15/58(25.9)	0.59	(0.30 – 1.17)	0.13	0.91	(0.39 – 2.12)	0.82
HCV genotype								
Non 3	196/232 (84.5)	45/58 (77.6)	1.00			1.00		
3	32/232 (13.8)	13/58(22.4)	1.76	(0.86 – 3.64)	0.12	1.43	(0.59 – 3.45)	0.43
TM6SF2 (genotype								
CC	215/232 (92.7)	46/58 (79.3)	1.00			1.00		
CT	17/232 (7.3)	12/58(20.7)	3.51	(1.18 – 10.47)	0.02	4.81	(1.67 – 13.87)	0.00

*Abbreviations*: *BMI* Body mass index, *HOMA-IR* homeostatic model assessment of insulin resistance

^a^Number of patients with the characteristic; ^b^Total number of patients with the outcome (no steatosis, steatosis, no advanced fibrosis or advanced fibrosis); ^c^In the case of continuous variables (age, BMI and HOMA-IR) means OR in one unit increase

After adjusting for age, gender, BMI, HOMA-IR, ethnicity/color, alcohol use, HCV genotype 3, and TM6SF2 genotypes, the association between PNPLA3 rs738409 GG genotype and advanced fibrosis remained significant in the recessive model (*p*=0.01) (Table 2). The gene TM6SF2 E167K variant (genotype CT) was also associated advanced fibrosis (*p*=0.00).

When analyzing the data, using the dominant and additive genetic inheritance model, we observed that patients with the GG genotype presented a higher risk of steatosis than patients with the CC genotype (*p*= 0.03, additive model) (Additional file 2). Patients with G allele presented higher risk of advanced fibrosis than those with the CC genotype (*p*=0.02, dominant model). However, patients carrying the GG genotype presented no greater risk of advanced fibrosis than those with the CC genotype (*p*=0.08, additive model) (Additional file 2). Regarding the TM6SF2 gene polymorphism, we observed that T-allele carriers presented a higher risk of fibrosis than patients with CC genotype (*p* = 0.01; dominant model).

## Discussion

This study assessed the influence of the rs738409 polymorphism of the PNPLA3 gene on hepatic steatosis and the degree of fibrosis among individuals diagnosed with chronic hepatitis C and observed that the prevalence rates of genotypes CC, CG, and GG of the PNPLA3 polymorphism were 45.9%, 21.7%, and 32.4%, respectively. We also observed that the GG genotype of the same polymorphism was associated with steatosis and advanced fibrosis. We also found an association between TM6SF2 polymorphism and advanced fibrosis. Our additional analysis reinforced the finding that the carriers of the rs847309 polymorphism genotype GG of the PNPLA3 gene are at increased risk of developing hepatic steatosis.

The influence of the rs738409 polymorphism on liver fat deposition was initially demonstrated in patients with NAFLD [5, 15, 16]. Sookoian et al. [16] also described the association between the polymorphism and fibrosis intensity, including nonalcoholic steatohepatitis. Later, this polymorphism was revealed to play a similar role in individuals with other liver diseases [17–19]. Regarding hepatitis C, several authors [7, 8, 20] have observed that the GG genotype is associated with a greater risk of steatosis and advanced fibrosis, whereas a study in Taiwan [21] observed that the G allele (CG + GG) was associated with the same events (i.e., the presence of steatosis and a greater degree of fibrosis). Our finding is in accordance with the data described above, as we also found that the G allele was an independent determinant of hepatic steatosis and advanced fibrosis.

PNPLA3, also referred to as adiponutrin, encodes a 481-amino-acid protein that contains a highly conserved patatin-like domain at the N terminus; in human tissues, its expression is highest in the liver, followed by skin and adipose tissue [22]. *In vitro* assays using recombinant PNPLA3 confirmed that the wild-type enzyme hydrolyzes emulsified TG and that the I148M substitution abolishes this activity. Expression of PNPLA3-I148M, but not wild-type PNPLA3, in cultured hepatocytes or in the livers of mice increased cellular TG contents [23]. The exact mechanism by which variation in PNPLA3 affects liver TG content is not known.

Related to the association between PNPLA3 and hepatic fibrosis, Pirazzi et al. [24] presented evidence of the mechanism involved in the association between PNPLA3 activity and hepatic fibrosis, regardless of the presence of steatosis. In that study, the authors demonstrated that PNPLA3 is highly expressed in human hepatic stellate cells (HSCs) suggesting a potential link between HSCs, retinoid metabolism, and PNPLA3 in determining the susceptibility to hepatic fibrosis.

Another finding of our study is that the E167K variant is an independent determinant of severity of fibrosis, as other researchers have also observed in NAFLD [25, 26] and hepatitis C [10]. In vivo [27, 28] and in vitro [27–29] studies suggest that TM6SF2 controls hepatic lipid efflux. Deletion or mutation of TM6SF2 results in reduced lipoprotein secretion (very-low-density lipoprotein [VLDL], TG, and apolipoprotein B [APOB]), which coincides with increased hepatocellular lipid droplet size and TG accumulation. The mechanism by which the E167K variant influences the severity of fibrosis is not known. In our study, rs58542926 TM6SF2 polymorphism was associated with advanced fibrosis independent of factors known to be associated with fibrosis, including the rs738409 PNPLA3 polymorphism.

The highest frequency of the PNPLA3 allele G was found in white Brazilians (45.6%), followed by black (35.7%) and *pardo* (34.9%) Brazilians. The prevalence of the GG genotype found in the study (32.4%) was higher than those found in European (2.2% to 12%) [7, 30–32] and Japanese studies (17.7% to 21.2%) [33–36]. However, the frequency of the G allele in white Brazilians was similar to that reported by Romeo et al. in American Hispanics (49.8%) [5]. Patients who considered themselves to be black had a higher prevalence of the G allele than the African Americans, according to Romeo et al [5].

According to the Brazilian census of 2010 [11] and to an autosomal DNA study [37], most Brazilians come from three large ancestral populations: European, African, and Brazilian Amerindians, in various combinations and varying degrees. Many Brazilians who define themselves as white may present African or Amerindian ancestors [38, 39], since in Brazil, individuals define themselves as white, black, or *pardo* based on skin color rather than on ancestry [11, 38, 39]. The mixture of ethnicities, even in patients who are considered white or black, could explain, at least in part, the different prevalence rates of the G allele of the rs738409 PNPLA3 gene polymorphism found in our study, although we could not locate the ethnic source of that difference.

Our study has limitations. The participants were recruited from a single tertiary care center linked to the university, limiting the number of participants in the study. Our results cannot be generalized to all patients with hepatitis C, although our case sample did not have a high proportion of patients with advanced liver disease (20.0%). In addition, the interpretation of our findings is also limited by the lack of a comparison group (e.g., patients with chronic liver disease of other etiologies or healthy volunteers) to help contextualize the events and characteristics present in patients with HCV.

Finally, despite these limitations, the data obtained should be added to the extant evidence regarding the roles that the rs738409 polymorphism of the PNPLA3 gene and the rs58542926 polymorphism of the TM6SF2 gene play in hepatic steatosis and advanced fibrosis among individuals with chronic hepatitis C. In addition, this study examined Brazilian patients, who (to the best of our knowledge) have not yet been studied from this perspective.

## Conclusions

Associations between the rs738409 polymorphism PNPLA3 gene genotype GG and hepatic steatosis and advanced fibrosis were observed among Brazilian patients with chronic HCV. In addition, we found that the rs58542926 polymorphism of the TM6SF2 gene is associated with advanced liver fibrosis independent of rs738409 polymorphism of the PNPLA3 gene.

Further studies are needed with comparison groups and larger numbers of patients to clarify the influence of the rs738409 polymorphism of the PNPLA3 gene and the rs58542926 polymorphism of the TM6SF2 gene (genotype CT) on hepatic steatosis and degree of fibrosis among individuals diagnosed with chronic hepatitis C.

## Additional files

Additional file 1: Clinical characteristics of patients stratified by ethnicity and genotype polymorphism at rs738409 PNPLA3. (DOCX 27 kb)

Additional file 2: Analysis of the association between genotype polymorphism at rs738409 PNPLA3 and steatosis and advanced liver fibrosis in patients with HCV infection according to the multivariate analysis in the dominant and additive model. (DOCX 26 kb)

## Abbreviations

γ-GT: Gamma-glutamyltransferase
ALT: Alanine aminotransferase
AST: Aspartate aminotransferase
BMI: Body mass index
CI: Confidence interval
DNA: Desoxyribunucleic acid
ELISA: Enzyme-linked immunosorbent assay
HBV: Hepatitis B virus
HCFMUSP: Hospital das Clínicas da Faculdade de Medicina da Universidade de São Paulo
HCV: Hepatitis C virus
HDL-C: High-density lipoprotein-cholesterol
HIV: Human immunodeficiency virus
HOMA-IR: Homeostatic model assessment
LDL-C: Low-density lipoprotein-cholesterol
OR: Odds ratio
PCR: Polymerase chain reaction
PNPLA3: Patatin-like phospholipase domain-containing 3 protein
RFLP: Restriction fragment length polymorphism
RNA: ribonucleic acid
SD: Standard deviation
SNP: Single-nucleotide polymorphism
SPSS: Statistical Software for the Social Sciences
TBE: Tris-Borate
TG: Triglycerides
TM6SF2: Transmembrane six superfamily member 2
VLDL-C: Very-low-density lipoprotein-cholesterol
