# Supplementary material for: The rs738409 polymorphism of the PNPLA3 gene is associated with hepatic steatosis and fibrosis in Brazilian patients with chronic hepatitis C
Source: BMC Infect Dis. 2017 Dec 19;17:780. doi: 10.1186/s12879-017-2887-6 (PMC5735770; doi:10.1186/s12879-017-2887-6)
Supplement: Supplementary file 1 — Clinical characteristics of patients stratified by ethnicity and genotype polymorphism at rs738409 PNPLA3. (DOCX 27 kb) [file 12879_2017_2887_MOESM1_ESM.docx]

**Additional file 1**.Clinical characteristics of patients stratified by ethnicity and genotype polymorphism at rs738409 PNPLA3

|  | **White** | | | ***Pardo*** | | | **Black** | | |
| --- | --- | --- | --- | --- | --- | --- | --- | --- | --- |
| **Characteristic** | **CC** | **CG** | **GG** | **CC** | **CG** | **GG** | **CC** | **CG** | **GG** |
| N | 100 | 46 | 80 | 23 | 10 | 10 | 10 | 7 | 4 |
| Gender (female/male) | 56/44 | 24/22 | 50/30 | 9/14 | 6/4 | 6/4 | 6/4 | 6/1 | 3/1 |
| Age (mean±SD) | 55.5±11.6 | 54.2±13.5 | 56.3±11.7 | 51.4±11.4 | 47.4±13.3 | 53.3±8.4 | 51.1±13.4 | 59±10 | 65±17.5 |
| BMI (mean±SD) | 27.4±6.7 | 26.5±4 | 27.9±7.4 | 25.7±4 | 27±5.1 | 25.4±4.4 | 26.3±4.1 | 23.6±3.6 | 26.5±4.5 |
| Glucose (mean±SD) | 87.5±32.7 | 88.7±36.4 | 90.2±26.6 | 103.4±59.6 | 89.9±27.4 | 139.2±161.8 | 91.3±23.1 | 144.1±134.2 | 90.8±24.3 |
| Insulin (mean±SD) | 12.2±7.2 | 25±77.4 | 13.9±9.7 | 11.4±9.1 | 23.3±25.9 | 16±6.1 | 13.7±7.4 | 8.7±3.9 | 13.9±12.3 |
| HOMA-IR(mean±SD) | 2.7±2.1 | 5.6±14.9 | 3.3±2.9 | 2.9±3.4 | 5.1±5.4 | 4.4±2.2 | 2.9±1.3 | 3.3±2.6 | 2.7±2.2 |
| ALT (mean±SD) | 66.7±56.3 | 57.7±36.5 | 63.7±52.8 | 55.5±39.5 | 47.3±34.6 | 80.8±46.3 | 47.6±27.2 | 61.3±64.9 | 48±17.1 |
| AST (mean±SD) | 49.9±39.1 | 47.3±35.6 | 51.9±39.3 | 41.8±23.8 | 42.5±35.5 | 81.1±66.4 | 43.3±22.2 | 47.9±38.6 | 39±10.7 |
| γ-GT (mean±SD) | 79.9±76.7 | 68.6±79.3 | 87.9±104.7 | 83.6±75.2 | 115.5±220.3 | 150.2±128.6 | 61.5±48.8 | 91.1±68.5 | 113.5±35.5 |
| Total cholesterol (mean±SD) | 169±40.3 | 172.8±31.4 | 176.2±38.3 | 180.4±46.7 | 160.3±22 | 167.5±26.9 | 174.1±35.4 | 210.1±84.4 | 211.3±27.5 |
| LDL-C (mean±SD) | 92.9±35.7 | 98.2±26.7 | 96.7±33.3 | 103.6±38.2 | 88.2±23.8 | 87.7±23.7 | 89±27.1 | 99.3±21.7 | 106.3±55.1 |
| HDL-C (mean±SD) | 53.8±17.3 | 52.6±15.4 | 55±19.3 | 55.7±16.3 | 53.2±15 | 56.3±16.2 | 69.1±27.7 | 62.4±15.4 | 83.5±40.8 |
| VLDL-C (mean±SD) | 23.2±14.2 | 22±14.3 | 26.4±24 | 21.1±8.9 | 18.9±7.6 | 23.5±9.4 | 16±7.4 | 48.4±83.3 | 21.5±5.9 |
| Triglycerides (mean±SD) | 109.4±62.5 | 107±68.5 | 112.7±77.2 | 105.7±44.8 | 94.2±37.5 | 117.4±47.3 | 89.3±36.8 | 137.3±139.4 | 101.5±19.7 |

Abbreviations: BMI, Body mass index; HOMA-IR, homeostatic model assessment of insulin resistance; ALT; alanine aminotransferase; AST, aspartate aminotransferase; γ-GT, gamma glutamyl transferase; LDL-C, low-density lipoprotein-cholesterol; HDL-C, high-density lipoprotein-cholesterol; VLDL, very-low-density lipoprotein-cholesterol
