# Supplementary material for: The rs738409 polymorphism of the PNPLA3 gene is associated with hepatic steatosis and fibrosis in Brazilian patients with chronic hepatitis C
Source: BMC Infect Dis. 2017 Dec 19;17:780. doi: 10.1186/s12879-017-2887-6 (PMC5735770; doi:10.1186/s12879-017-2887-6)
Supplement: Supplementary file 2 — Analysis of the association between genotype polymorphism at rs738409 PNPLA3 and steatosis and advanced liver fibrosis in patients with HCV infection according to the multivariate analysis in the dominant and additive model. (DOCX 26 kb) [file 12879_2017_2887_MOESM2_ESM.docx]

**Additional file 2.** Analysis of the association between genotype polymorphism at rs738409 PNPLA3 and steatosis and advanced liver fibrosis in patients with HCV infection according to the multivariate analysis in the dominant and additive model

|  | **Dominant (CC vs CG/GG)** | | | **Additive (CC vs GG)** | | |
| --- | --- | --- | --- | --- | --- | --- |
| **Variables** | **OR^a^** | **95% CI** | ***P*** | **OR^b^** | **95% CI** | ***P*** |
| **Steatosis present (≥5%)** |  |  |  |  |  |  |
| PNPLA3 (rs738409) | 1.79 | (1.01 – 3.20) | 0.05 | 1.99 | (1.05 – 3.75) | 0.03 |
| Age (years) | 1.04 | (1.02 – 1.06) | 0.00 | 1.03 | (1.00 – 1.06) | 0.05 |
| Sex (male) | 0.61 | (0.35 – 1.06) | 0.08 | 0.43 | (0.22 – 0.85) | 0.02 |
| BMI (kg/m2) | 1.10 | (1.03 – 1.17) | 0.00 | 1.07 | (1.00 – 1.14) | 0.06 |
| Ethnicity/color (*pardo*) | 0.78 | (0.36 – 1.68) | 0.52 | 0.73 | (0.29 – 1.81) | 0.49 |
| Ethnicity/color (black) | 0.74 | (0.26 – 2.09) | 0.57 | 0.85 | (0.26 – 2.80) | 0.78 |
| HOMA-IR | 1.00 | (0.96 – 1.84) | 0.99 | 1.25 | (1.06 – 1.46) | 0.01 |
| Alcohol (>20 g/day) | 1.36 | (0.75 – 2.44) | 0.31 | 1.56 | (0.79 – 3.10) | 0.20 |
| HCV (genotype 3) | 2.07 | (0.99 – 4.31) | 0.05 | 1.95 | (0.84 – 4.54) | 0.12 |
| TM6SF2 (genotype CT) | 2.21 | (0.91 – 5.35) | 0.08 | 2.21 | (0.74 – 6.62) | 0.16 |
|  |  |  |  |  |  |  |
| **Advanced fibrosis (F3/4)** |  |  |  |  |  |  |
| PNPLA3 (rs738409) | 2.32 | (1.13 – 4.76) | 0.02 | 2.11 | (0.91 – 4.90) | 0.08 |
| Age (years) | 1.08 | (1.04 – 1.11) | 0.00 | 1.08 | (1.03 – 1.13) | 0.00 |
| Sex (male) | 0.22 | (0.10 – 0.47) | 0.00 | 0.40 | (0.16 – 0.96) | 0.04 |
| BMI (kg/m2) | 1.03 | (0.97 – 1.10) | 0.29 | 1.03 | (0.96 – 1.10) | 0.44 |
| Ethnicity/color (*pardo*) | 2.37 | (0.91 – 6.16) | 0.08 | 4.55 | (1.55 – 13.37) | 0.01 |
| Ethnicity/color (black) | 1.48 | (0.38 – 5.74) | 0.57 | 3.56 | (0.80 – 15.81) | 0.10 |
| HOMA-IR | 1.02 | (0.98 – 1.07) | 0.30 | 1.33 | (1.13 – 1.57) | 0.00 |
| Alcohol (>20 g/day) | 1.02 | (0.44 – 2.36) | 0.97 | 1.23 | (0.47 – 3.19) | 0.68 |
| HCV (genotype 3) | 1.60 | (0.67 – 3.79) | 0.29 | 3.01 | (1.09 – 8.28) | 0.03 |
| TM6SF2 (genotype CT) | 3.87 | (1.36 – 11.06) | 0.01 | 1.58 | (0.33 – 7.53) | 0.57 |

Abbreviations: BMI, Body mass index; HOMA-IR, homeostatic model assessment of insulin resistance

^a^ Reference for OR: patients with CC genotype

^b^ Reference for OR: patients with CC genotype
